# Pharmacodynamic Effects of a 6-Hour Regimen of Enoxaparin in Patients Undergoing Primary Percutaneous Coronary Intervention (PENNY PCI Study)

**DOI:** 10.1055/s-0038-1657768

**Published:** 2018-06-06

**Authors:** Wael Sumaya, William A. E. Parker, Rebekah Fretwell, Ian R. Hall, David S. Barmby, James D. Richardson, Javaid Iqbal, Zulfiquar Adam, Kenneth P. Morgan, Julian P. Gunn, Annah E. Mason, Heather M. Judge, Christopher P. Gale, Ramzi A. Ajjan, Robert F. Storey

**Affiliations:** 1 Department of Infection, Immunity and Cardiovascular Disease, Sheffield Medical School, University of Sheffield, Sheffield, United Kingdom; 2 South Yorkshire Cardiothoracic Centre, Northern General Hospital, Sheffield Teaching Hospitals NHS Foundation Trust, Sheffield, United Kingdom; 3 Leeds Institute of Cardiovascular and Metabolic Medicine, University of Leeds, Leeds, United Kingdom

**Keywords:** STEMI, primary PCI, enoxaparin, P2Y _12_ inhibition, stent thrombosis

## Abstract

Delayed onset of action of oral P2Y _12_ inhibitors in ST-elevation myocardial infarction (STEMI) patients may increase the risk of acute stent thrombosis. Available parenteral anti-thrombotic strategies, to deal with this issue, are limited by added cost and increased risk of bleeding. We investigated the pharmacodynamic effects of a novel regimen of enoxaparin in STEMI patients undergoing primary percutaneous coronary intervention (PPCI). Twenty patients were recruited to receive 0.75 mg/kg bolus of enoxaparin (pre-PPCI) followed by infusion of enoxaparin 0.75 mg/kg/6 h. At four time points (pre-anti-coagulation, end of PPCI, 2–3 hours into infusion and at the end of infusion), anti-Xa levels were determined using chromogenic assays, fibrin clots were assessed by turbidimetric analysis and platelet P2Y _12_ inhibition was determined by VerifyNow P2Y12 assay. Clinical outcomes were determined 14 hours after enoxaparin initiation. Nineteen of 20 patients completed the enoxaparin regimen; one patient, who developed no-reflow phenomenon, was switched to tirofiban after the enoxaparin bolus. All received ticagrelor 180 mg before angiography. Mean (± standard error of the mean) anti-Xa levels were sustained during enoxaparin infusion (1.17 ± 0.06 IU/mL at the end of PPCI and 1.003 ± 0.06 IU/mL at 6 hours), resulting in prolonged fibrin clot lag time and increased lysis potential. Onset of platelet P2Y _12_ inhibition was delayed in opiate-treated patients. No patients had thrombotic or bleeding complications. In conclusion, enoxaparin 0.75 mg/kg bolus followed by 0.75 mg/kg/6 h provides sustained anti-Xa levels in PPCI patients. This may protect from acute stent thrombosis in opiate-treated PPCI patients who frequently have delayed onset of oral P2Y _12_ inhibition.

## Introduction

Dual oral anti-platelet therapy is essential in patients presenting with ST-elevation myocardial infarction (STEMI) and undergoing primary percutaneous coronary intervention (PPCI). 1

Potent P2Y _12_ inhibition, with prasugrel or ticagrelor, resulted in improved outcomes following acute coronary syndrome. 2 3 Although these two agents provide rapid platelet inhibition in stable patients, 4 5 their onset of action can be delayed in patients undergoing PPCI, particularly those pre-treated with opiates such as morphine. 6 7 8 9 10 This negative interaction might increase the risk of acute stent thrombosis, 11 necessitating initial parenteral anti-thrombotic treatment to cover the period prior to P2Y _12_ inhibitor absorption.

Available parenteral therapies to deal with this issue include glycoprotein IIb/IIIa inhibitors (GPIs) 12 or intravenous P2Y _12_ inhibition with cangrelor. 13 However, these options have several limitations. GPIs increase the risk of major bleeding events and routine use has resulted in worse outcomes in several trials. 14 15 16 17 Cangrelor has some advantages: It inhibits platelet P2Y _12_ receptors within 2 minutes and has rapid offset of action over 1 hour after cessation of infusion. 13 However, it has primarily been used as a 2-hour infusion and this might not be sufficient in patients with more than 2 hours delay in the onset of oral P2Y _12_ inhibition. Moreover, the very rapid offset of action may pose a risk should the infusion be interrupted. GPIs and cangrelor are costly, another factor that is likely to limit widespread routine use.

Enoxaparin, a low-molecular-weight heparin, targets factor Xa and, to a lesser extent, factor IIa (thrombin). 18 It has a half-life of 1.5 to 2 hours when given intravenously 19 and provides more predictable anti-thrombotic effect compared with unfractionated heparin (UFH), negating the need for monitoring. 20 A bolus dose of 0.5 or 0.75 mg/kg of enoxaparin can be given to support the PPCI procedure. 1 In addition to blocking coagulation through the formation of fibrin, heparins inhibit platelet activation by opposing the action of thrombin, 21 which activates platelets via protease-activated receptors 1 and 4. 22 UFH paradoxically increases platelet reactivity to soluble agonists such as adenosine diphosphate, representing potentially undesirable effects in patients undergoing PCI, whereas therapeutic concentrations of enoxaparin have minimal impact on this. 23 24

In the ATOLL trial, 25 a bolus dose of enoxaparin 0.5 mg/kg (with additional 0.25 mg/kg for prolonged procedures) in patients undergoing PPCI resulted in comparable results to a bolus dose of UFH (50–70 IU/kg with GPIs or 70–100 IU/kg without GPIs). However, over 75% of all patients received GPIs. It may, therefore, be more appropriate to use a higher bolus dose of anti-coagulant when GPIs are not used, as was previously acknowledged when studying the combination of UFH with GPI. 26

We hypothesized that an infusion of enoxaparin (0.75 mg/kg/6 h) following a bolus dose of 0.75 mg/kg would provide sufficient and sustained anti-thrombotic effects to bridge treatment with oral P2Y _12_ inhibition in STEMI patients undergoing PPCI, without the need for GPIs or cangrelor. In this study, we aimed to assess the pharmacodynamic effects of this regimen.

## Materials and Methods

### Study Design

This was a single-centre, single-arm, open-label pharmacodynamic study. All study patients provided informed verbal consent pre-PPCI followed by written consent as soon as possible following PPCI according to a protocol approved by the local research ethics committee and the Medicines and Healthcare Products Regulatory Agency, UK. The trial was registered at http://clinicaltrials.gov (unique identifier: NCT03146858).

In vitro studies of the inhibitory effects of enoxaparin on thrombin-induced platelet activation were also performed to support the rationale for the clinical study. Detailed methods and results of these in vitro studies are reported in the Supplementary Material (available in the online version).

### Study Population and Intervention

Patients presenting with STEMI and accepted for PPCI were recruited if they met the inclusion and exclusion criteria as detailed in the Supplementary Material (available in the online version). All patients were pre-treated with ticagrelor 180 mg on arrival to hospital and before angiography. Following angiography, an intra-arterial bolus of enoxaparin 0.75 mg/kg was administered and an intravenous infusion of 0.75 mg/kg/6 h was started. For the infusion, enoxaparin (0.75 mg/kg) was added to 250 mL of normal saline and started through an infusion pump at a rate of 42 mL/h. We planned to stop the infusion at 3 hours in cases of significant renal impairment (estimated glomerular filtration rate [eGFR] < 30 mL/min/h). Only in cases of no-reflow was GPI use permitted and enoxaparin infusion was to be stopped if a GPI was administered. Concomitant use of other anti-coagulants was prohibited and patients were excluded if they had received UFH or another anti-coagulant prior to the procedure.

Blood was collected into citrated tubes at four time points: pre-anti-coagulation, at the end of PPCI, 2 to 3 hours into infusion and at the end of infusion. Clinical outcomes were determined at least 14 hours after initiation of infusion. Outcomes of interest were any thrombotic or bleeding complications.

### Pharmacodynamic Measurements

Tissue factor was obtained from Stago, recombinant tissue plasminogen activator (tPA) from Technoclone, sodium chloride from Sigma-Aldrich and calcium chloride dehydrate and Tris from Fisher Scientific.

Plasma was derived and stored at –80°C until analysis. The primary outcome measure was anti-Xa levels at the three time points during the infusion. Anti-Xa levels were measured using chromogenic assays utilizing the commercially available Coamatic kit (Chromogenix) and Sysmex CS-5100 analyser.

Fibrin clot properties were assessed using a validated turbidimetric assay. 27 28 Permeation buffer solution (100 mM NaCl, 50 mM Tris, pH 7.4) was used for dilution. A total of 25 μL of plasma (in duplicates) was mixed with 75 μL of the activation and lysis mix (83 ng/mL tPA, 10 pM tissue factor, 17 mM calcium chloride; final concentrations) at 37 **°** C. Multiskan FC (Thermo Scientific) was used to read plates every 30 seconds at 340 nm until lysis of all samples. Fibrin clot lag time, maximum turbidity (a measure of fibrin clot density, expressed as arbitrary units [AU]) and lysis time (time taken for turbidity to drop by 50%) were recorded.

Platelet P2Y _12_ inhibition was assessed in whole blood using the VerifyNow P2Y12 system at least 20 minutes post-sampling. P2Y12 reactivity units (PRUs) and VerifyNow % inhibition (determined using the thrombin receptor-activating peptide channel results as estimated baseline response) were recorded. Evidence of P2Y _12_ inhibition was defined as VerifyNow % inhibition ≥ 20%.

Thromboelastometry was performed on a subset of patients recruited during working hours. This was performed according to the manufacturer's instructions using a ROTEM delta analyser and in-tem S reagents (Tem Innovations). Clotting time, clot formation time, amplitude at 10 minutes and maximum clot firmness were recorded.

### Statistical Analysis

A sample size of 19 with an *α* of 0.05 gives this study over 90% power to detect 25% drop in anti-Xa levels during the 6-hour infusion. Continuous data are presented as mean ± standard error of the mean or median (interquartile range), as appropriate. Categorical data are presented as numbers and proportions. One-way analysis of variance (ANOVA) with Dunnett's multiple comparison tests was used for assessment of continuous variables. Effect of anti-emetic treatment on P2Y _12_ inhibition was assessed using two-way ANOVA. Results with *p* -values of < 0.05 were considered statistically significant. Statistical analyses were performed using GraphPad Prism 7 for Mac OS X.

## Results

### Study Population

Twenty patients were recruited with median age of 67 years ( Table 1 ). Eighty per cent were males, 45% had anterior STEMI, all had PPCI through the radial artery approach and one patient required an intra-aortic balloon pump. All patients received a loading dose of 180 mg ticagrelor pre-PPCI, administered as whole tablets. Nineteen patients completed the full enoxaparin regimen and were included in the pharmacodynamic analyses. No patient had an eGFR < 30 mL/min. One patient was switched to tirofiban due to no-reflow phenomenon after enoxaparin bolus but prior to starting enoxaparin infusion.

**Table 1:** Baseline, procedural and treatment characteristics

Variable	Value
Age (y, median [IQR])	67 (57–77)
Male sex (%)	16/20 (80)
Caucasian race (%)	18/20 (90)
Anterior STEMI (%)	9/20 (45)
Smoking (%)	12/20 (60)
Hypertension (%)	9/20 (45)
Diabetes mellitus (%)	3/20 (15)
Chronic kidney disease (%)	3/20 (15)
Previous acute coronary syndrome (%)	3/20 (15)
Previous PCI (%)	2/20 (10)
Treatment with opiates (morphine/diamorphine)	18/20 (90)
Pain to balloon time (min, median [IQR])	205 (131–364)
Call to balloon time (min, median [IQR])	146 (114–179)
Door to balloon time (min, median [IQR])	47 (41–65)
Stent length (mm, median [IQR])	22 (14–30)
Stent diameter (mm, median [IQR])	3.5 (3–4)
Ticagrelor loading to 1st blood sampling (min, median [IQR])	20 (10–40)
Ticagrelor loading to 2nd blood sampling (min, median [IQR])	60 (50–95)
Ticagrelor loading to 3rd blood sampling (min, median [IQR])	185 (175–220)
Ticagrelor loading to 4th blood sampling (min, median [IQR])	405 (390–425)

Abbreviations: IQR, interquartile range; PCI: percutaneous coronary intervention; STEMI: ST-elevation myocardial infarction.

Note: Chronic kidney disease is defined as estimated glomerular filtration rate (eGFR) < 60 mL/min.

### Pharmacodynamic Assessments

**Fig. 1:**
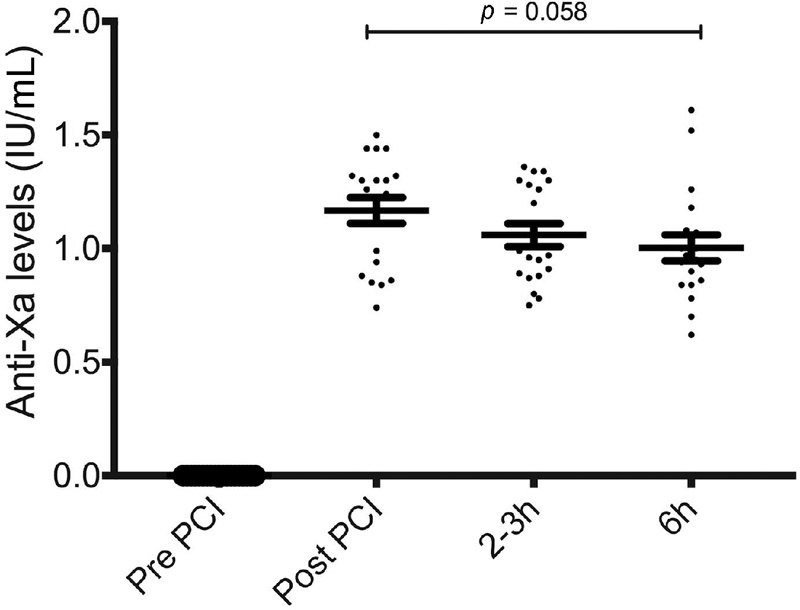
Anti-Xa levels. A scatter plot demonstrating anti-Xa levels throughout the studied time points ( *n* = 19). Variance during the infusion was assessed using one-way analysis of variance (ANOVA). Six hours versus 2 to 3 hours; *p* = 0.6 and 6 hours versus post-PCI; *p* = 0.09. Error bars represent mean ± standard error of the mean (SEM).

Anti-Xa levels peaked at the end of PPCI (1.17 ± 0.06 IU/mL) and were subsequently sustained both 2 to 3 hours into infusion (1.01 ± 0.05 IU/mL) and at the end of infusion (1.003 ± 0.06 IU/mL) ( Fig. 1 ).

**Fig. 2:**
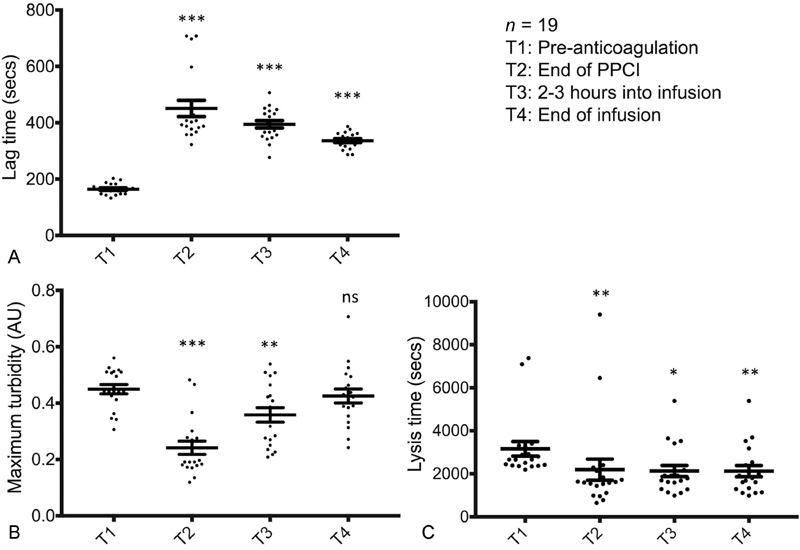
The effects of enoxaparin on fibrin clot properties. Scatter plots demonstrating fibrin clot properties pre- and throughout treatment with enoxaparin. Error bars represent mean ± standard error of the mean (SEM). AU, arbitrary unit; PPCI, primary percutaneous coronary intervention. *** denotes *p* < 0.001, ** denotes *p* < 0.01, * denotes *p* = 0.01; all compared with baseline (T1) and calculated using Dunnett's multiple comparisons tests.

Pre-anti-coagulation, the turbidimetric assay demonstrated rapid fibrin clot formation with a lag time of 165 ± 4.4 seconds. This significantly prolonged at the end of PPCI (431 ± 29 seconds; *p* = 0.0001 vs. baseline) and remained prolonged during the infusion at 2 to 3 hours (395 ± 13 seconds; *p* = 0.0001) and at the end of infusion (337 ± 6.6 seconds; *p* = 0.0001) ( Fig. 2A ).

Fibrin clot maximum turbidity measured was 0.45 ± 0.02 AU pre-anti-coagulation. This significantly dropped at the end of PPCI (0.24 ± 0.02 AU; *p* = 0.0001 vs. baseline) and gradually increased during the infusion (0.36 ± 0.03 AU; *p* = 0.0012 vs. baseline) returning to baseline at the end of infusion (0.43 ± 0.02 AU; *p* = 0.7 vs. baseline) ( Fig. 2B ).

Pre-anti-coagulation, lysis time measured 3,161 ± 339 seconds. This significantly dropped by the end of PPCI (2,192 ± 489 seconds; *p* = 0.001 vs. baseline) and remained low during the infusion (2,128 ± 256 seconds; *p* = 0.014 vs. baseline) and at the end of infusion (2,124 ± 259 seconds; *p* = 0.009 vs. baseline) ( Fig. 2C ).

### Platelet P2Y _12_ Inhibition

All patients were P2Y _12_ inhibitor naive on presentation. Time from ticagrelor loading to different time points is summarized in Table 1 .

**Fig. 3:**
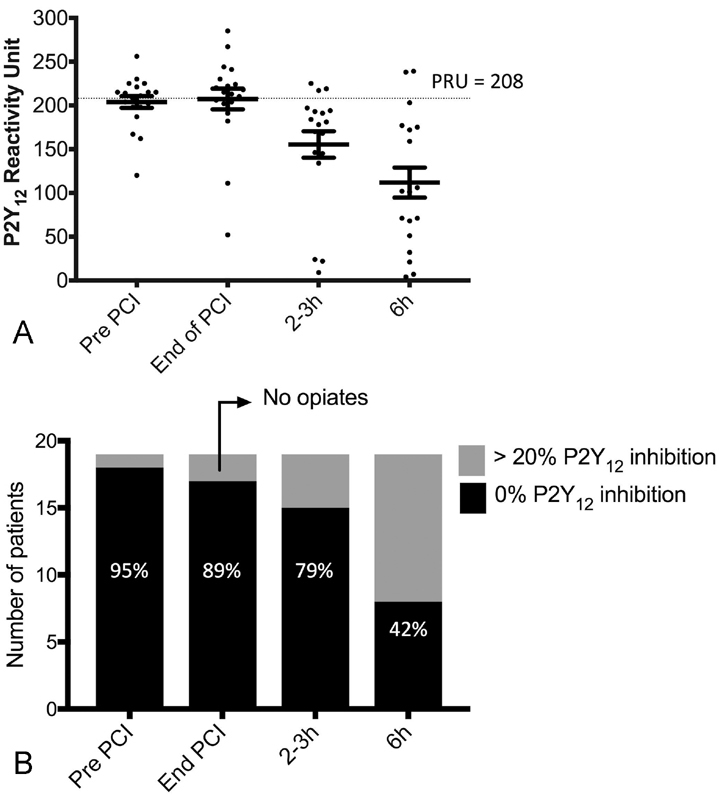
Platelet P2Y _12_ inhibition. ( **A** ) Scatter plot demonstrating the corresponding P2Y _12_ reaction units throughout the studied time points. Error bars represent mean ± standard error of the mean (SEM). ( **B** ) Bar graph highlighting the number of patients with 0% inhibition as determined by VerifyNow. Two patients did not receive opiates and had > 20% inhibition by the end of PCI (as indicated by arrow).

Using a PRU cut-off of < 208 for evidence of P2Y _12_ inhibition, 12/19 patients at the end of PPCI, 3/19 at 2 to 3 hours and 2/19 patients at 6 hours had no P2Y _12_ inhibition. The two opiate-free patients had levels < 208 by end of PPCI ( Fig. 3A ).

Using VerifyNow % inhibition, all but the two opiate-free patients had 0% inhibition by end of PPCI ( Fig. 3B ). Poor P2Y _12_ inhibition remained common in morphine-treated patients throughout the 6-hour infusion with 8/19 patients still having 0% inhibition at 6 hours despite treatment with anti-emetics (metoclopramide ± ondansetron) in all but 3 opiate-treated patients. Treatment with anti-emetics did not affect P2Y _12_ inhibition (interaction *p* = 0.26). Only one patient vomited following ticagrelor's administration and was subsequently re-loaded with ticagrelor shortly after PPCI.

### Thromboelastometry

Thromboelastometry was performed in a subset of 11 patients. Only clotting time was significantly prolonged throughout the infusion, whereas the other parameters were insensitive to the effects of enoxaparin ( Supplementary Fig. S2 , available in the online version).

### Clinical Outcomes

None of the patients suffered any thrombotic or bleeding complication during the follow-up period (14–24 hours post-PPCI).

## Discussion

We have studied the pharmacodynamic profile of a novel enoxaparin regimen in STEMI patients undergoing PPCI and have shown that it results in sustained anti-Xa levels throughout the 6-hour infusion. This regimen positively modulated fibrin clot properties, prolonging lag time and improving lysis potential, indicating the formation of less thrombotic clots. 29 30 Inefficient lysis is an independent predictor of cardiovascular death following MI and so this regimen might help to improve prognosis. 31 The universal prolongation of lag time following enoxaparin was prominent and consistent throughout, which may provide a useful functional measure of response to this therapy. Interestingly, fibrin clot studies provided more consistent data compared with thromboelastometry, suggesting a higher sensitivity of this method for detecting the anti-thrombotic effects of enoxaparin.

Delayed absorption of oral P2Y _12_ inhibitors in STEMI patients is increasingly recognized by interventional cardiologists as a stent thrombosis risk. Crushing tablets have been attempted as a way to accelerate absorption of oral P2Y _12_ inhibitors. 32 33 34 35 However, results were marginal and delayed absorption in a proportion of STEMI patients remains a risk. Similarly, increasing ticagrelor's loading dose did not overcome the problem. 36 Therefore, parenteral therapies are likely to be needed to protect a large majority of patients.

This novel enoxaparin regimen has the advantage of being simple and easy to implement in an emergency situation. Enoxaparin is also widely used for many other indications with good tolerability. Moreover, it is inexpensive and therefore likely to offer substantial cost savings when compared with GPIs or cangrelor.

We should acknowledge the limitations of our study. This is a single-arm pharmacodynamic study and clinical outcomes are only reported as pilot outcomes. Larger randomized studies are needed to establish the safety and efficacy of the studied enoxaparin regimen in STEMI patients undergoing PPCI. Furthermore, platelet P2Y _12_ inhibition was only studied with one methodology although we have found this methodology to be most discriminatory in the assessment of platelet P2Y _12_ inhibition. 37 Our study has sufficient power to detect 25% drop in anti-Xa levels at the two time points. The degree of variance in anti-Xa levels is of borderline significance ( *p* = 0.058) but levels at the end of infusion remained therapeutic, resulting in sustained improvement in fibrin clot lysis potential. A high percentage of patients had poor platelet inhibition by the end of infusion, which raises the possibility that the duration of infusion might not be sufficient. However, enoxaparin's half-life is approximately 2 hours and more P2Y _12_ inhibition is highly likely to be established by 8 hours after loading dose of ticagrelor. This requires further assessment in future work.

## Conclusion

A bolus dose of enoxaparin (0.75 mg/kg) followed by an infusion of 0.75 mg/kg/6 h results in sustained pharmacodynamic effects throughout the infusion in STEMI patients undergoing PPCI. The efficacy and safety of this regimen should be evaluated in larger studies.

## Notes

**What is known about the topic?**

- Platelet P2Y _12_ inhibition is often delayed in opiate-treated STEMI patients undergoing PPCI.
- This delay in platelet inhibition may increase the risk of stent thrombosis.
- Strategies to manage this risk are needed.

**What does this paper add?**

- A novel regimen of enoxaparin (0.75 mg/kg bolus followed by 0.75 mg/kg/6 h) in STEMI patients undergoing PPCI results in sustained anti-Xa levels.
- This regimen positively modulates fibrin clot properties, prolonging fibrin clot lag time, reducing fibrin clot density and enhancing lysis potential.
- This regimen may offer an inexpensive solution to the delayed absorption of oral P2Y _12_ inhibitors but future studies are needed to confirm safety and efficacy.
