# Supplementary material for: Pharmacodynamic Effects of a 6-Hour Regimen of Enoxaparin in Patients Undergoing Primary Percutaneous Coronary Intervention (PENNY PCI Study)
Source: Thromb Haemost. 2018 Jun 6;118(7):1250–6. doi: 10.1055/s-0038-1657768 (PMC6202933; doi:10.1055/s-0038-1657768)
Supplement: Supplementary file 1 — Supplementary Material [file 10-1055-s-0038-1657768-s180086.pdf]

Supplementary Material

In Vitro Studies of the Anti-Platelet Action of Enoxaparin

Materials: Blood was taken into tubes containing 3.13% (wt/vol) tri-sodium citrate dihydrate. Fluo-4 AM, probenecid (water soluble) and $CaCl_2$ were from Fisher Scientific (Loughborough, UK). Thrombin was from Sigma-Aldrich (Gillingham, UK). PAR1 agonist peptide (SFLLRNPNDKYEPF) was synthesized by Almac Group Ltd (Craigavon, UK). Enoxaparin (Clexane) was from Sanofi-Aventis (Guildford, UK). Antithrombin 3 (ATIII) was from Haematologic Technologies Inc (Vermont, United States). ATIII control was 50% glycerol/water (vol/vol). HEPES Tyrodes buffer was 129 mmol/L NaCl, 8.9 mmol/L $NaHCO_3$, 2.8 mmol/L KCl, 0.8 mmol/L KH_2PO_4, 5.6 mmol/L dextrose and 10 mmol/L HEPES.

Methods: Blood was obtained from healthy volunteers who had not taken any anti-platelet medication in the previous 10 days and anti-coagulated with 3.13% (wt/vol) tri-sodium citrate at a ratio of 1:9. Volunteers gave written informed consent and all studies were performed with local ethics board approval. Blood samples were centrifuged at $200 \times g$ to produce platelet-rich plasma (PRP). PRP was labelled with fluo-4 AM (3 µmol/L) in the presence of probenecid (2.5 mmol/L) for 30 minutes at 37°C. Fluo-4 AM labelled PRP was pre-incubated with enoxaparin (0.25–2 anti-Xa U/mL) for 30 minutes. Small aliquots of labelled PRP (2 µL) were diluted 1:250 with HEPES-tyrodes buffer containing a physiological concentration of ATIII (0.125 mg/mL) or control, 1 mmol/L calcium chloride and enoxaparin at the concentrations indicated in the 'Results' section. Baseline fluorescence was measured for 10 seconds using Becton Dickinson LSRII flow cytometer (Oxford, UK). Thrombin (0.5 U/mL) was then added directly to the vial and fluorescence was monitored for a further 2 minutes. Area under the curve for increase in median fluorescence was determined. Statistical analysis was performed using the GraphPad Prism software (version 5.0).

Results: Thrombin induces a rapid calcium mobilization that peaks within 5 seconds and decays back towards baseline fluorescence levels within 2 minutes (►**Supplementary Fig. S1A**, available in the online version). Preliminary studies were performed to look at the interaction between enoxaparin and ATIII. Enoxaparin alone partially inhibited the peak calcium mobilization and enhanced the rate of calcium re-uptake into platelets; ATIII alone similarly resulted in a small degree of inhibition but, when added in addition to enoxaparin, greatly enhanced the inhibition seen with enoxaparin alone (►**Supplementary Fig. S1A**, available in the online version). Increasing concentration of enoxaparin in the presence of ATIII progressively inhibited thrombin-induced platelet calcium mobilization, with enoxaparin 1 to 2 U/mL almost abolishing the increase in fluorescence (►**Supplementary Fig. S1B**, available in the online version).

Interpretation: Enoxaparin effectively inhibits thrombin-induced platelet activation at therapeutic concentrations in the presence of a physiological concentration of ATIII.

Inclusion and Exclusion Criteria for the PENNY PCI Study

Inclusion Criteria

1. Age ≥ 18.
2. Confirmation of the diagnosis of ST-elevation myocardial infarction (STEMI) on the basis of a classical history, electrocardiographic (ECG) changes and angiographic findings.
3. Pre-treatment with either ticagrelor or prasugrel.
4. Intention to proceed with primary percutaneous coronary intervention (PPCI).
5. Feasibility to obtain informed verbal consent pre-PPCI.

Exclusion Criteria

1. Active bleeding that cannot be controlled by local measures.
2. Pregnant patients.
3. Patients with end-stage renal failure requiring renal replacement therapy.
4. Known thrombocytopenia (platelet count $< 100{,}000$/µL).
5. Known history of intra-cranial haemorrhage.
6. Known current treatment with oral anti-coagulants.
7. Known history of major surgery or trauma or history of GI/GU haemorrhage within the last month.
8. Known intra-cranial malignancy or aneurysm.
9. Known allergy to enoxaparin.
10. Known hypersensitivity to benzyl alcohol.
11. Patients with acute bacterial endocarditis.
12. Active gastric or duodenal ulceration.
13. Inability to easily understand verbal information given in English for any reason.
14. Inability to give informed consent due to either temporary or permanent mental incapacity.
15. Current participation, or participation within the last month, in an interventional clinical trial.

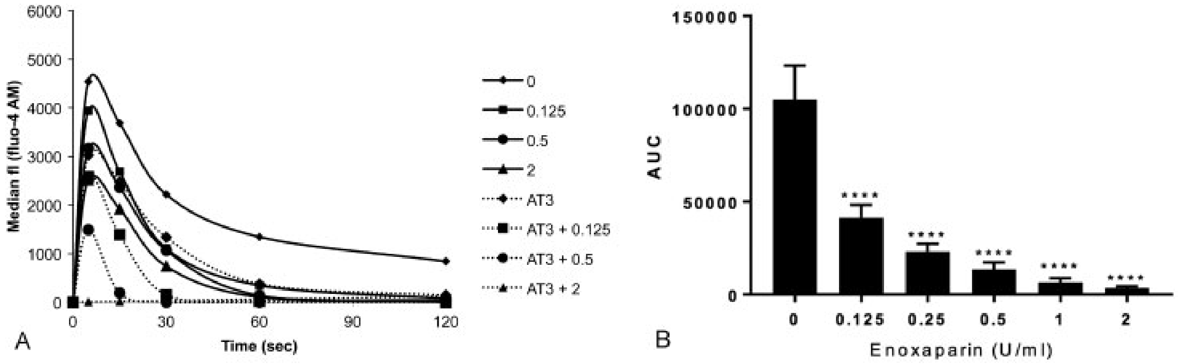

Supplementary Fig. S1 Inhibition of thrombin 0.5 U/mL-induced calcium mobilization by enoxaparin (0–2 U/mL). Platelets were labelled with fluo-4 AM and the increase in median fluorescence induced by 0.5 U/mL thrombin was measured by flow cytometry over 2 minutes assessing (**A**) the response in the absence or presence of 0.125 mg/mL anti-thrombin III (AT3) and enoxaparin 0, 0.125, 0.5 and 2 U/mL and (**B**) area under the curve (AUC) with enoxaparin 0 to 2 U/mL. Data are mean ± standard error of the mean (SEM), $n = 6$. Difference in the AUC was determined using analysis of variance (ANOVA). $^{****}p < 0.0001$.

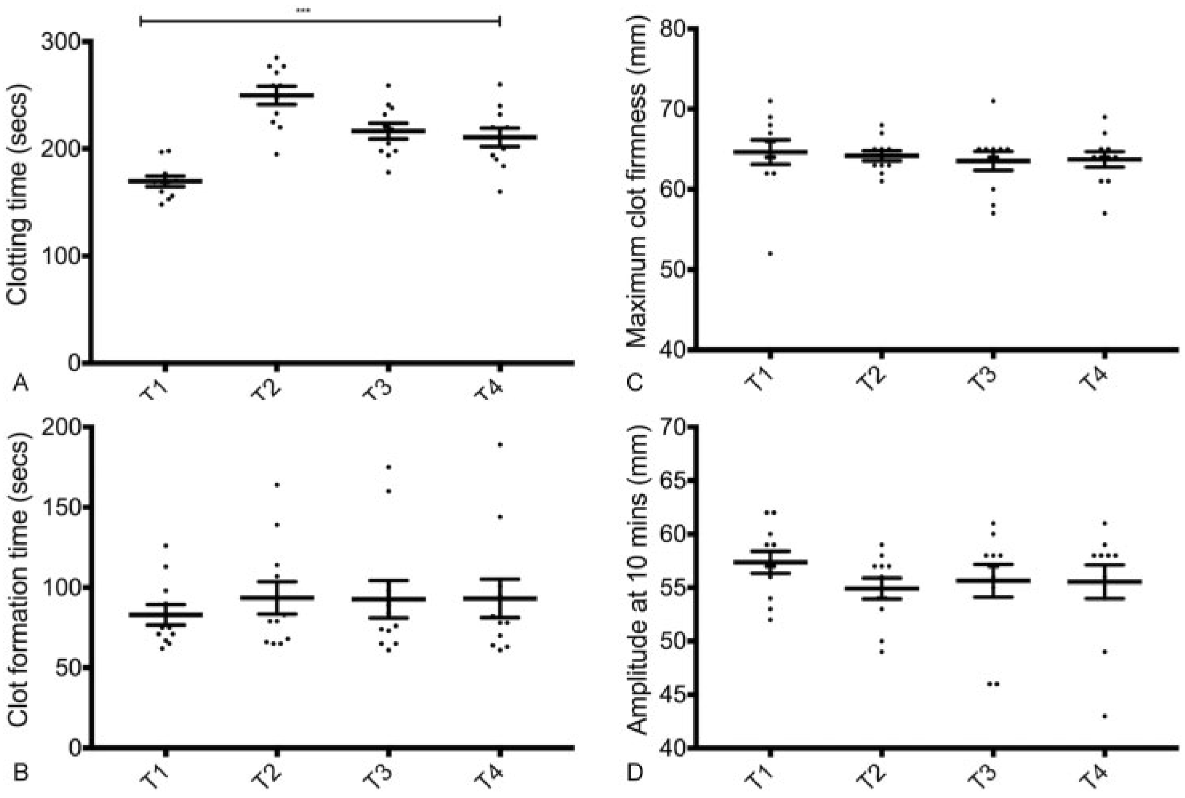

Supplementary Fig. S2 The effects of enoxaparin on thromboelastometry. Scatter plots of all thromboelastometry measurements ($n = 11$). T1: pre-anti-coagulation; T2: end of primary angioplasty; T3: 2 to 3 hours into infusion; T4: end of infusion. *** denotes $p < 0.0001$, calculated by analysis of variance (ANOVA).
